# Changing food waste regimes in Africa’s transition to export-oriented production: the case of Tanzanian avocado

**DOI:** 10.1007/s10460-024-10699-5

**Published:** 2025-01-27

**Authors:** Jonas Cromwell, Stephen Whitfield, Claire Helen Quinn, Megan Kathleen Blake

**Affiliations:** 1 School of Food Science and Nutrition, University of Leeds, Leeds, UK; 2 School of Earth and Environment, University of Leeds, Leeds, UK; 3 Department of Geography, University of Sheffield, Sheffield, UK

**Keywords:** Food waste regimes, Food loss, Food waste, Institutions, Materiality, Practices

## Abstract

African nations are increasingly focusing on exporting high-value crops. However, a major challenge exists: high rates of food waste within supply chains. The problem is often seen as a technological issue—a lack of proper infrastructure and coordination creates inefficiencies. This research takes a different perspective, focusing on social relations within the supply chain. It uses the concept of “food waste regimes” to understand the underlying structures, relationships, and systems that cause food waste, with a focus on Tanzania’s avocado trade. The goals of the research are to: (1) Identify the factors contributing to food waste within Tanzania’s avocado supply chains, particularly in the context of export-oriented production; (2) Explore how these factors change as production shifts towards exports; (3) Analyse the fairness (equity) of how waste burdens are distributed among those involved. We adopted a “follow the thing” approach, combining interviews and observations across both domestic and export avocado supply chains in Tanzania. The research reveals that interactions between various aspects of the supply chain—practices, physical properties of the product (avocado perishability), and established institutions—influence where food waste occurs and who shoulders the burden of that waste. The research exposes how unequal power dynamics between participants lead to some actors bearing a disproportionate amount of the risk and cost of food waste. By taking a social relations approach, this research highlights that tackling food waste and social inequality are intertwined issues. The paper suggests potential areas for future research and intervention to address these interconnected challenges.

## Introduction

The sustainable transformation of food systems has become a high-profile global agenda in response to a range of environmental and societal challenges (UNFSS 2021). The transition towards increased production of high-value food commodities for commercial export is at the centre of a range of national economic development and agricultural sector policy priorities across Africa. This transition has been underpinned by significant donor-driven development of and private-investment in large commercial land acquisitions, agri-technological innovation, processing and transport infrastructure, trade regulation development and harmonisation, and more. The value of agricultural exports from the African continent saw a 6% annual growth between 1999 and 2019 (Johnson et al. 2023) and the Malabo Declaration set out the aim of tripling intra-African trade in agricultural commodities between 2014 and 2025 (African Union Commission 2014). Over the past decade, the Tanzanian government has prioritised transitioning to export-oriented production and markets through policy instruments, particularly the Agricultural Sector Development Programme (ASDP I & ASDP II). Launched in 2015, ASDP II is a ten-year initiative building on ASDP I, aiming to transform the agricultural sector (crops, livestock, and fisheries) by enhancing productivity, commercialization, and smallholder farmer income. This transformation seeks to improve livelihoods, food and nutrition security, and contribute to the country’s GDP by expanding exports to international markets. ASDP II aligns with the vision and principles outlined in the Tanzania Agriculture and Food Security Investment Plan (TAFSIP).

There is an increasingly recognised risk that food system transformations perpetuate, rather than tackle, embedded social inequalities within food systems, resulting in all-too-familiar patterns of winners and losers at local scales or the dark side of transformation (Blythe et al. 2018). The premise of this paper is that food system transitions—from domestic to export production, and particularly those relating to market engagement, have equity implications that can be illustrated in the form of waste accumulations, burdens, and risks. The approach of the paper is to look at these implications through the lens of food waste.

A recent report by WWF-UK (2021), which focuses on farm-level losses and waste, identified primary production as a major hotspot for global food losses. The report estimates that global food loss and waste on farms (including early stages of supply chains) amounts to 1.2 billion tonnes per year, representing 15.3 per cent of global agricultural production. In Africa, the African Union Commission (AUC) have set the target to halve current levels of food waste (estimated at 37% of production) from post-harvest loss by 2025 under the Malabo Declaration’s commitment to ending hunger in Africa by 2025 (AUC 2018). In Tanzania, efforts to reduce food loss and waste have been on going and the National Post-Harvest Management Strategy (2019–2029), aims to reduce post-harvest losses to sufficiently contribute to food and nutrition security and the economy (URT—PHMS 2019). With approximately 900,000 people (13% of a population of 7.1 million people) in 21 district councils of Mainland Tanzania experiencing high levels of acute food insecurity, reducing food waste calls for an urgent attention (URT-IPC 2023).

In the past decades there has been a significant growth in the production and export of avocados in Tanzania, catalysed by a combination of donor-driven development programmes, such as the USAID Tanzania Agriculture Productivity Programme and export-oriented national agricultural policy (ASDP I & II). A recent report by MARKUP (2020) indicated that Tanzania’s share of the global avocado market has increased from 0.1 to 0.4% since 2013. In Tanzania, there are key differences between domestic and export avocado production systems in terms of supply chain organisation, stakeholders, marketing infrastructure, cultivars grown, and farming practices, all of which result in different regimes of waste production. Fresh fruits are the most wasted food commodity and a significant contributor to total food loss in global food supply chains (WWF-UK 2021).

In Tanzania, losses in the horticulture sub-sector are estimated at around 40–50% (URT 2019). A study of the mango value chain in the Morogoro region found that losses ranged between 48 and 60% along the chain (Msogoya and Kimaro 2011). A recent study by Ekka and Mjawa (2020) on the role of Tanzania’s Horticulture Association (TAHA) in reducing post-harvest loss (PHL) in the sector estimated 40% PHL for fruits and vegetables in the domestic supply chains compared to 10% in the export-oriented chains. In the domestic avocado production, losses during harvesting range from 5 to 30%, with an additional 30–40% at the wholesale/retail stage due to mechanical damage and pests or diseases (Juma et al. 2019; Cromwell 2022). In the export avocado sector, losses are estimated at 30–50% of the total production for smallholders and some large-scale farmers; and 10–20% for companies or commercial farms due to fruits not meeting quality standards. Similar post-harvest loss rates (35% in domestic chain and 15% losses in export chain) have been reported in Kenya’s avocado supply chains (Snel et al. 2021a; 2021b). However, policy interventions to reduce food loss and waste often frame the food loss and waste problem as ‘inefficiency and technological inadequacy within supply chains’—whether relating to agricultural management practices or lack of post-harvest infrastructure (Gille 2013, p. 39). Therefore, policy interventions place emphasis on technological improvements in production, storage, and distribution (Sheahan and Barrett 2017; Affognon et al. 2015).

This research stems, in part, from a desire to challenge the dominant view that food losses and waste in the global South are due to inefficiencies in agricultural, storage, and infrastructural systems. It does so by recognising the inherent power in agricultural production systems and the relational role of institutions, practices, and materiality in producing and distributing waste. There have been calls to take a more social relations approach to researching food losses and waste (Evans et al. 2013; Alexander et al. 2013; Gille 2013; Evans 2011, 2012). Here we take a step away from the dominant question of “how much waste is there” to ask “what”, “how”, and “why” losses and waste occur (Moreno et al. 2020; Evans 2018; Watson and Meah 2013). Drawing on Gille’s (2013) concept of global food waste regimes, our objective is to understand what underlying structures, relationships and systems drive food waste within avocado supply chains in Tanzania, how these change with a shift towards export-oriented production, and what the equity implications are.

## Background—a food waste regimes framework

The food waste regimes concept, according to Gille (2013, p. 29), “consists of social institutions and conventions that do not only determine what wastes are considered valuable but also regulate their production and distribution”. Approaching food loss and waste from this perspective allows us to consider the role of institutions, materiality, and practices in food waste production. In this way, food waste can be viewed as socially produced—a consequence of the relationship between the materiality of the food commodity, the practices through which it is produced and exchanged, and the formal and informal institutions that govern and direct supply chain interactions.

### Institutions

Institutions as used here, broadly means “the rules of the game in a society; more formally, they are the humanly devised constraints that shape human interaction” (North 1992, p. 477). Institutions could be formal rules or informal rules (Keefer and Shirley 2000; Williamson 2002). The approach assumes that actors calculate the best course of action to maximise their interests within a specific institutional arrangement (Ostrom 1986), where the institutional arrangement refers to “a set of rules or agreements governing the activities of a specific group of people pursuing a certain objective” (Eaton et al. 2008, p. 10; Williamson 1998). In the context of this research, formal institutions may include, for example, the rules and constitutions governing producer organisations and contract farming—the rules for joining and leaving and sanctions applied when rules are broken (van der Maden et al. 2021; Eaton et al. 2008; De Putter et al. 2007). Food safety rules and regulations, quality standards, trade rules, and certification schemes that determine how the food crop must be grown, harvested, transported, processed, and traded are all forms of formal institutions (Gereffi et al. 2005; Henson and Reardon 2005; Busch 2000, 2004; Busch and Lawrence 2005; Freidberg 2004). Informal institutions, could include, for example, cultural values and norms that shape the agronomic practices of growing the avocados, gendered roles in soil management, weeding and harvesting and more. However, there is little understanding of the ways that social relations within institutional arrangements operate in the Tanzania context to create waste both in domestic and export avocado production. While several studies (e.g., van der Maden et al. 2021; Eaton et al. 2008) have examined institutional arrangements within the Tanzanian horticulture sector, they fail to extend such analysis to understanding how such social relations lead to loss and waste generation. The same is true for other studies that have examined institutional arrangements and social relations in horticulture production in Nigerian and Ghana (Omeihe et al. 2021; Amoako 2019; Lyon and Porter 2009; Lyon and Porter 2009). Moreover, there is little understanding and evidence of how the new institutional arrangements associated with a transition towards increased export production impact loss and waste across supply chains.

### Materiality

While notions of materiality are broad among different academic disciplines, what is common is the problematization of how materiality has been framed within the social scientific scholarship. Schatzki (2010) argues that for many theorists, materiality is understood to mean physicality (e.g., the materiality of the world is its physical constituents and properties). However, materiality can mean something broader than physical properties. In this sense, materiality includes nature, manmade objects, our bodies, and in broader sense, the ways space is organised around us, and the concrete practices and the technologies we employ in our everyday lives (Gille 2014, 2016). Thus, materiality considers sets of related entities—humans, artefacts, organisms, and things of nature (Schatzki 2002, 2010).

Until now, there has been little attention on using the material relational approach to understand processes of agricultural waste in general, and specifically food loss and waste in agricultural production systems. An exception is Anna Krzywoszynska’s (2013) work on the relational materiality of bio-wastes from the Italian winemaking industry. She calls for consideration to be given to ‘materiality, temporality, and spatiality’ if excess bio-waste materials are to be integrated into sustainable rural landscapes (ibid). When applied to analysis of food waste, a materiality approach helps to bring to light the roles of the natural environment, variety of the avocado grown, cultural norms, values, and framings about avocado trees/fruits, and the conditions of their production and consumption in food losses and waste generation.

### Practices

According to Schatzki (2002, p. 87), ‘a practice is a temporally evolving, open-ended set of doings and sayings.’ The doing and saying involve rules, specific ways of understanding, knowledge of how things work and how to use things, but also the state of emotion (Schatzki 2002; Reckwitz 2002). Many studies within agri-food systems have engaged with theories of practice to ‘increase understanding of the transformation and changes in farming practices’ (Huttunen and Oosterveer 2017, p. 191). Such engagement has primarily emerged in the arena of sustainable transition or transformation in agricultural practices—e.g., sustainable fertilization usage, organic farming, agroecology, (Freyer and Bingen 2012; Genus et al. 2021; Sahakian et al. 2017); urban agriculture (Jansma and Wertheim-Heck 2021); understanding farmers’ routinized crop protection practices (Kaiser and Burger 2022); and agricultural extension systems (Paschen et al. 2021). While such analysis improves our understanding of farming practices in relation to transformation, mainly from conventional agriculture to sustainable agriculture and crop protection practices, it falls short of illuminating how practices lead to food losses and waste in agricultural production.

A better understanding of the underlying causes of farm loss and waste (including pre-harvest losses and losses in early stages of supply chains) requires integrating the concepts of materiality, institutions and the practices and the interrelationship and interaction between them. For example, how do institutions and materiality (including the crop being grown) shape the enacted agronomic practices? And in what ways does that impact losses and waste? These are among some of the gaps in the literature that we try to address. Such understanding will move the food loss and waste debate beyond just placing it at the doorstep of technological inadequacy (Gille 2013).

## Case study—avocado supply chains in Tanzania

**Fig. 1:**
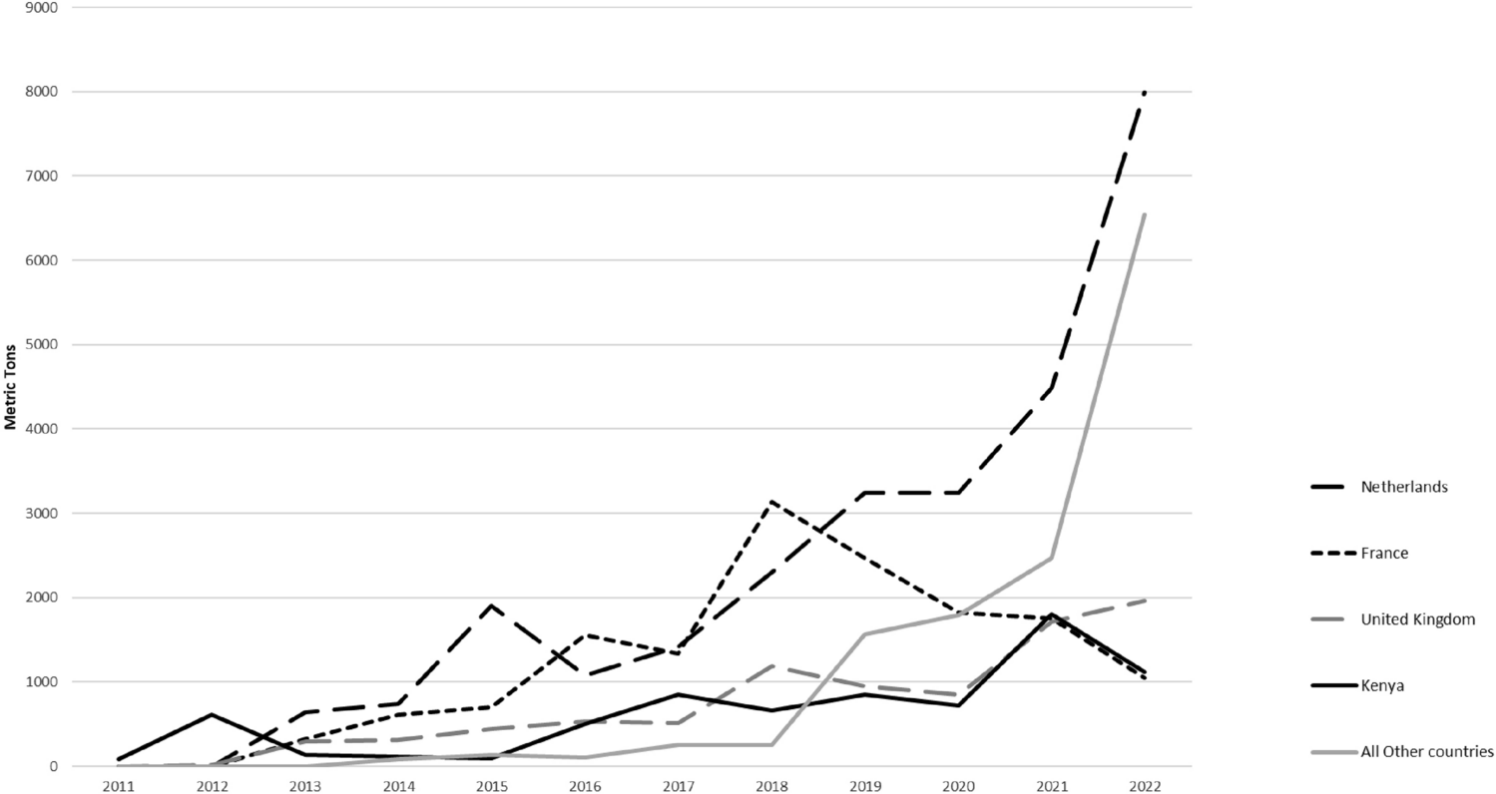
Tanzania’s Avocado Export Destinations in metrics tons from 2011 to 2022. Source: Data from International Trade Centre—Trade Map database (2024)

According to the National Horticulture Development Strategy and Action Plan (NHDS & AP 2021–2031), the horticulture industry is the fastest-growing industry within Tanzania’s agricultural sector, with an annual growth rate of 9 to 12% and contributing 38% of total foreign income from agriculture production (URT 2021b). Horticulture export value has grown from USD 412 million in 2015 to USD 779 million in 2019 and is the second biggest foreign exchange earner (after tea) as government policies focus on transitioning from domestic to export production (URT 2021b; George 2022). The NHDS & AP aims to increase production by 40% from the current production levels of 7,560,010 tons in 2019/2020 (URT 2021b). Tanzania is Africa’s third-largest avocado producer, after South Africa and Kenya, and ranks 19th globally in exports. Annually, it produces around 190,000 tons of avocados (Tanzanian Trade Development Authority 2019). The global fresh avocado market, valued at $6.5 billion in 2020, is projected to reach $19.9 billion by 2026 (Statista 2024); therefore, Tanzania has a role to play in meeting this global demand (Fig. 1).

Avocados have been grown in Tanzania for the last hundred years, first introduced by the German missionaries in the early 1890s in Kilimanjaro and Mbeya regions. In Tanzania, there are two distinct avocado production systems and value chains with different cultivars, distribution systems and stakeholders (Cromwell 2022). While domestic avocado production is mainly subsistence and for the domestic market, export avocado production is predominantly for the export market. The domestic avocado is cultivated for various purposes, including food, firewood, shade for coffee trees, timber, and animal feed (using leaves for fodder and fruit as feed), and as part of agroforestry systems (Fernandes et al. 1985). Unlike cash crops such as coffee, tea, and cashew, avocado did not receive substantial research and support during the colonial era and after independence, which hindered the industry’s development (Coulson 2013). The market and supply chain only developed in the late 1990s when traders began transporting them to Dar es Salaam and other cities, from Kilimanjaro (Cromwell 2022), compared to fruits like papaya, banana, and oranges, which have longer history of long-distance trading (see Lynch 1992). This delay was due to widespread ownership of avocado trees in the production areas, lack of awareness about their nutritional benefits, and low economic value (Cromwell 2022).

### The transition from domestic to export-oriented production

**Fig. 2:**
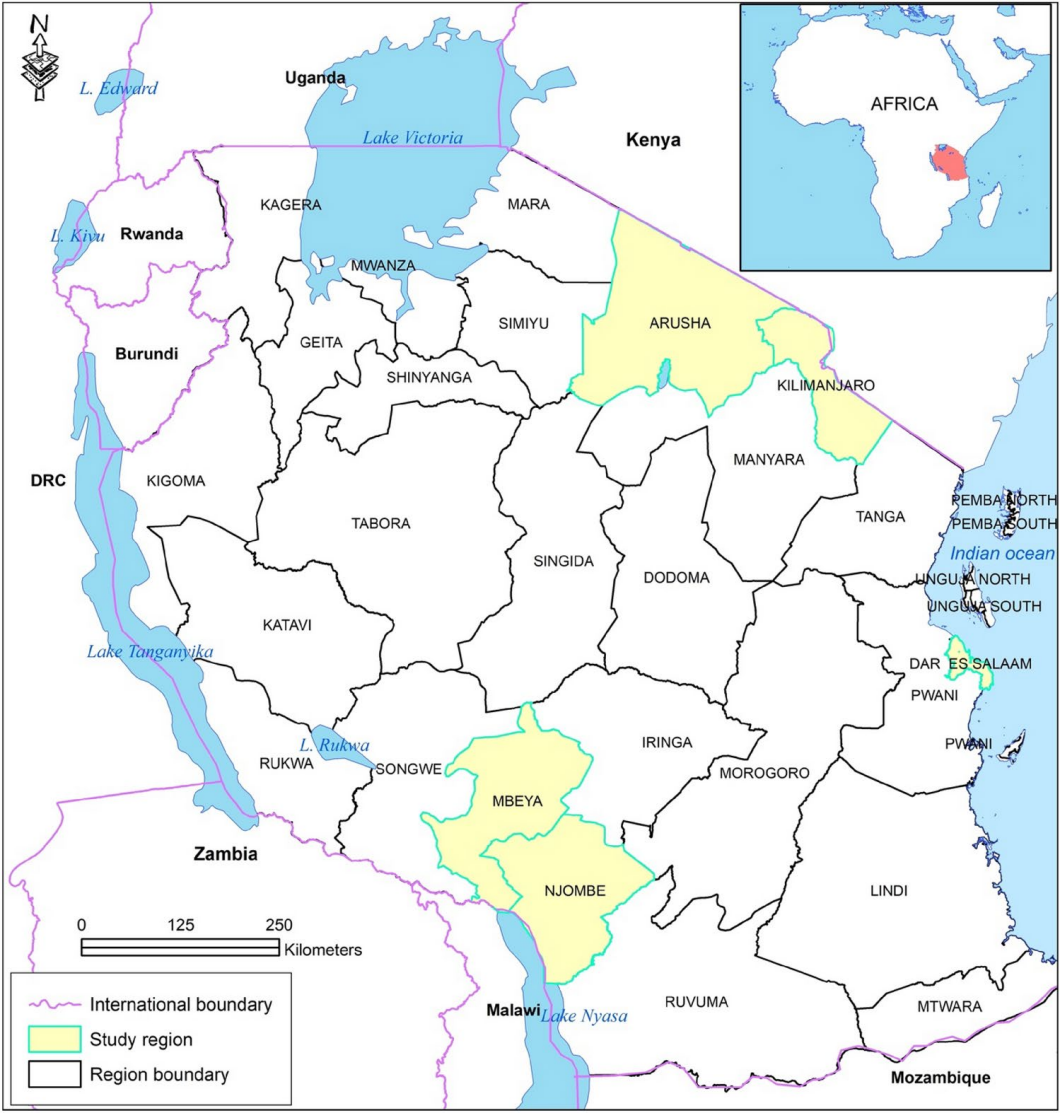
Map of Tanzania Showing study regions in the Northern Highlands (NH) and Southern Highlands (SH) and Dar es Salam

While no effort has been made to commercialise the production of domestic avocado cultivars, in recent decades there has been a shift towards commercial production of export-oriented cultivars, particularly Hass. The first attempt to introduce commercial export varieties was in the early 1990s with donor support (Mwakalinga 2014). About 16 cultivars of germplasm were imported from Israel and the USA for trials within this program. The Hass and Fuerte varieties showed promise for good production and were recommended for dissemination. However, there was poor uptake of the new varieties in the Northern Highlands (NH), especially in Kilimanjaro and Arusha regions (see Fig. 2), since consumers did not like them (Mruma 2013). In the Southern Highlands (SH)—Mbeya and Njombe regions, there was a moderate success. About 9000 Fuerte and Hass seedlings were distributed to smallholder farmers in selected villages, but mainly for subsistence production (Mruma 2013).

The transition from domestic to commercial export production did not start until 2007, with significant private sector investments backed with considerable donor support to get smallholder farmers involved. The start of commercial avocado production can be attributed to lower coffee prices in the late 1990s, coupled with structural adjustment programs in the 70 s and 80 s, increasing global demand for avocados, and shifts in government agricultural policy to attract private sector investment in export-oriented commercial farming (primary policies like ASDP I & II and programs such as Kilimo Kwanza and SAGCOT). For instance, the USAID funded Tree Crop Project (USD 800,000) in 2007 focused on temperate fruit production, including Hass avocado. Between 2009 and 2014 under the Tanzania Agriculture Productivity Programme (USAID-TAPP), subsidised Hass avocado seedlings were supplied to smallholders (Mruma 2013). In the last decade, there has been significant growth in production due to the expansion by export company farms (e.g., Africado Ltd), strong growth in independent commercial farmers, new production regions (particularly in the SH), and a steadily growing number of smallholders and expansion in smallholder production.

FruiTrop (2019) estimated that in 2018, between 1200 and 1400 hectares of land was under export avocado production, with an estimated yearly growth of 300 to 400 hectares in the cultivated area. With this growth, it is projected that avocado exports could reach around 15,000 to 20,000 metric tonnes by 2023. Cromwell (2022) noted that there are over 10,427 farmers engaged in export avocado production cultivating an estimated 2,271 hectares. The majority of farmers (99.33%) are smallholders with 83.30% owning between 1 and 100 trees (about an acre), while 16.03% own between 100 and 1200 trees (1–10 acres) and are responsible for 1143 hectares. Commercial farmers (10 acres and above), who are less than 1% (0.67%) account for nearly half of the total land under avocado production.

Tanzania exported its first avocados (100 MT) to Europe in 2008 through Kenya. Since then, there has been tremendous growth in export. According to export figures from International Trade Centre, between 2013 and 2022, export volume increased from 1393 MT to 18,668 MT, representing an increase of over 1340% (ITC, 2024). Similarly, export earnings have increased steadily from a little under half a million USD (0.42 million USD) in 2011 to over 20 million USD in 2022 (ITC 2024). Between 2021 and 2022, export value increased by 109%, from nearly 11 million USD to nearly 21 million USD. In 2023, Tanzania’s avocado exports reached 26,826 MT, valued at 77 million USD (Daily News 2024).

Although exports of other key vegetables (onions, tomatoes, green beans, leeks, potatoes, peppers) and spices (cloves) have also seen growth in the past decade (George 2022), unlike avocados, there has not been steady growth. For instance, the export value for potatoes has peaked at below USD 1 million per year, while export value for tomatoes has witnessed a decline from USD 981,000 in 2013 to just USD 74,000 in 2018. Cloves, even though they account for 87% of current spice exports with an export volume averaging 1,051 MT per year, are still below the peak volume of 3,795 MT per year recorded between 2011 and 2014 (George 2022).

Regarding export destinations for Tanzanian avocados, the EU is the largest market. In 2022, the Netherlands alone accounted for 43% of Tanzania’s total export volume, with an export value of USD 8.76 million. The UK is the second-largest destination, accounting for 10.52% of the export volume. Besides Europe, Kenya is a major export destination in Africa due to many Kenyan exporters sourcing fruits from Tanzania. The normalization of phytosanitary rules between South Africa and Tanzania in 2021 has also made South Africa a significant importer, especially during the low season (Dec-Mar) in South Africa (TAHA 2022). Additionally, new emerging markets in China, the United Arab Emirates, Russia, and India provide diversified opportunities for export farmers. Both India and China have signed free trade agreements with Tanzania for horticultural products.

## Methods

### The ‘follow the thing’ approach

The approach taken to research in this study is inspired by the ‘follow the thing’ methodology used by Cook (2004, 2006) and Cook and Harrison (2007) which tells the biographies of the food commodity being followed. The follow the thing approach draws on Appadurai’s (1988, p. 5) call to follow global commodities to understand the social, cultural, political, and economic relations behind food commodities, and Marcus’ (1995) multi-sited ethnography approach, which argues that it is no longer possible to study globalised and transnational processes in a single place. Earlier studies using ‘follow the thing’ work backwards from the commodity through “assembling of pre-figured point of sale [of the] commodity” (Gregson et al. 2010, p. 5), and tracing it to the point of its origin (Hulme 2017). However, there have been calls to attend to flows ‘down’ the value chain (Gregson et al. 2010). In this study, the direction of ‘following’ started from the farm to the wholesale markets (domestic supply chain) and the packhouse (export supply chain). This approach was embedded within a qualitative case study design to build an in-depth contextual understanding of the case—changing food waste regimes in Tanzania’s avocado production systems (Yin 2018; Creswell 2013). Such a novel design approach provides a unique advantage for this research in two ways:

First, following the avocados from the farms to local markets, wholesale markets, and to packhouses offered the best opportunity to understand how the interrelations between institutions, materiality and practices manifest in the production system to produce losses and waste. It allowed the different actors and stakeholders who engage with the avocado through its journeys to be part of the research participants, thereby giving a richer contextual understanding of how waste production occurs and the social relations within the avocado production system.

Overall, the design strategy afforded multiple forms of data collection (interviews, go-along participant observation, informal conversations, and secondary data) (Creswell 2013), which aided triangulation of findings and a comprehensive understanding of the differences and inconsistencies in participants’ accounts (DeWalt and DeWalt 2011). Besides, the analytic strategy provided a ‘thick description’ of each case and themes within the case (Geertz 1973) by illustrating everyday experiences of avocado waste generation with textured accounts drawn from multiple voices, locations, and scales.

### Data collection

The research was conducted across several sites in Tanzania—categorised into three main areas: the NH (Kilimanjaro and Arusha regions), SH (Mbeya and Njombe regions), and Dar es Salaam (Fig. 2). The NH and SH were selected because there are differences in production scale and institutional arrangement between farmers and exporters/buyers, while Dar es Salaam was selected as a major destination for domestic avocado production. The fieldwork was conducted between 2018 and 2021 over several extended and short visits. The main data collection occurred over one extended visit (May to September 2018) and two short visits (March–April 2019 and July–August 2019), and several follow-up conversations with key stakeholders during the COVID-19 pandemic. The fieldwork was planned to coincide with the main harvesting seasons for domestic and export avocados and organised into two phases.

Phase 1 of the data collection (May–September 2018 and March–April 2019) took place in six districts in the NH. Go-along participant observations (harvesting and selling) and interviews were conducted with various participants in both domestic and export supply chains (Tables 1, 2 and 3). Phase 2 of the fieldwork (July–August 2019), took place in the Rungwe and Njombe town districts in the SH (Tables 2 and 3).

**Table 1:** Go-along participant observation (domestic avocado supply chain)

Nature of go-along	No. of ‘go-along’. (days’)	Type of participant	No. of traders (followed)	Wholesale markets	District	Region
Harvesting & selling	11	local brokers	8	Sanya Juu Mwika Lawati Mamsera	Siha Moshi rural Rombo	Kilimanjaro
Selling	7	Wholesalers	3	Sanya Juu Mamsera Mwika	Siha Moshi rural Rombo	Kilimanjaro
Selling	2	Wholesalers	1	Ilala Boma	Ilala	Dar es Salaam
Selling	3	Agents	5	Ilala Boma Temeke Stereo Mabibo	Ilala Temeke Ubungo	Dar es Salaam
Total	23		17

**Table 2:** Go along participant observation (export supply chain)

Nature of go-along	No. of go-along (in days’)	Type of participants	District	Region
Harvesting	2	Commercial farmer	Meru	Arusha
Harvesting	4	Smallholders	Hai/Siha/Rombo	Kilimanjaro
Harvesting/collection	6	Field officers	Rombo/Siha/Meru	Kilimanjaro Arusha
Grading/processing/packaging	2	Packhouse manager/supervisors	Siha	Kilimanjaro
	1	Packhouse manager/supervisors	Rungwe	Mbeya
Total go-alongs	15

**Table 3:** Interview participant domestic and export supply chains

Domestic avocado Supply Chain			
Category of participants	No Male	No Female	Total
Farmers (smallholders)	7	13	20
Fruit pickers	6	2	8
Local brokers	4	14	18
Packers	5		5
Wholesalers	3	5	8
Agent traders (Dar es Salaam)	6	2	8
Key informants*	4		4
Total participants	35	36	71
Export avocado supply chain (NH&SH)			
Smallholders (micro-scale; 1–100 avocado trees)	36	7	43
Smallholders (small-scale; 100–1200 avocado trees)	11	3	14
Large-scale farmers (above 1200 avocado tress)	4		4
Commercial export-producer companies	4		4
Nursery owners	5		5
Field officers (export companies/processors)	6	3	9
Out-grower manager	2		2
Farmer groups (association/cooperatives leaders)	4		4
Packhouse supervisors	2	2	4
Export managers/packhouse managers	3		3
Technical managers/farm managers	5		5
MD of export companies	2		2
Other key informants (Global GAP external auditor, TAHAfresh, SAGCOT, Agriculture officials, Ward/Village officials)	9		9
Total participants	93	15	108

Key informants (District agricultural officer, Ward extension officer, Director of local NGOs)

In all, 85 in-depth interviews were conducted with farmers, comprising domestic avocado farmers (20) and different groups of export avocado farmers (65). While female farmers (13) dominated domestic avocado production, male farmers (both small and large scale) dominated export avocado production. Historically, the domestic avocado is considered a food crop and not a cash crop, and men tend to dominate cash crop production.

A total of 34 traders were interviewed, consisting of local brokers, wholesalers, and agents (Table 3). Besides the traders, shorter interviews with fruit pickers, packers, and helpers (13 in total), helped to triangulate data gathered with traders and farmers, especially in understanding the grading criteria used in the market and packaging practices.

Go-along participant observations were conducted with actors in both domestic (17 days) and export supply chains (15 days). The go-along allowed for several direct observations and informal conversations with helpers, packers, and other traders, and to witness broader trading practices and norms in the markets beyond the traders that we were observing.

### Data analysis

All interviews, including informal conversations during go-alongs and follow-ups, were audio-recorded and transcribed verbatim. Using practice framing, the interview data were coded thematically using an inductive approach, in which themes were allowed to emerge through careful reading of the data (Charmaz 2014). All transcribed material was coded, and a point of saturation was reached when new codes ceased to emerge through continued analysis. NVivo 12 software was used in organising and coding the data.

## Results

### The structure of domestic and export avocado supply chains

Supply chains associated with both domestic and export avocados are multiple and multifaceted, involving different combinations of, and interactions between, producers, buyers, agents, regulators, and retailers. Here, we map out these supply chains and actors, drawing broad comparisons between domestic and export supply chains and providing context to the subsequent analysis of where waste is generated and how it is distributed across these chains.

#### Domestic supply chain

**Fig. 3:**
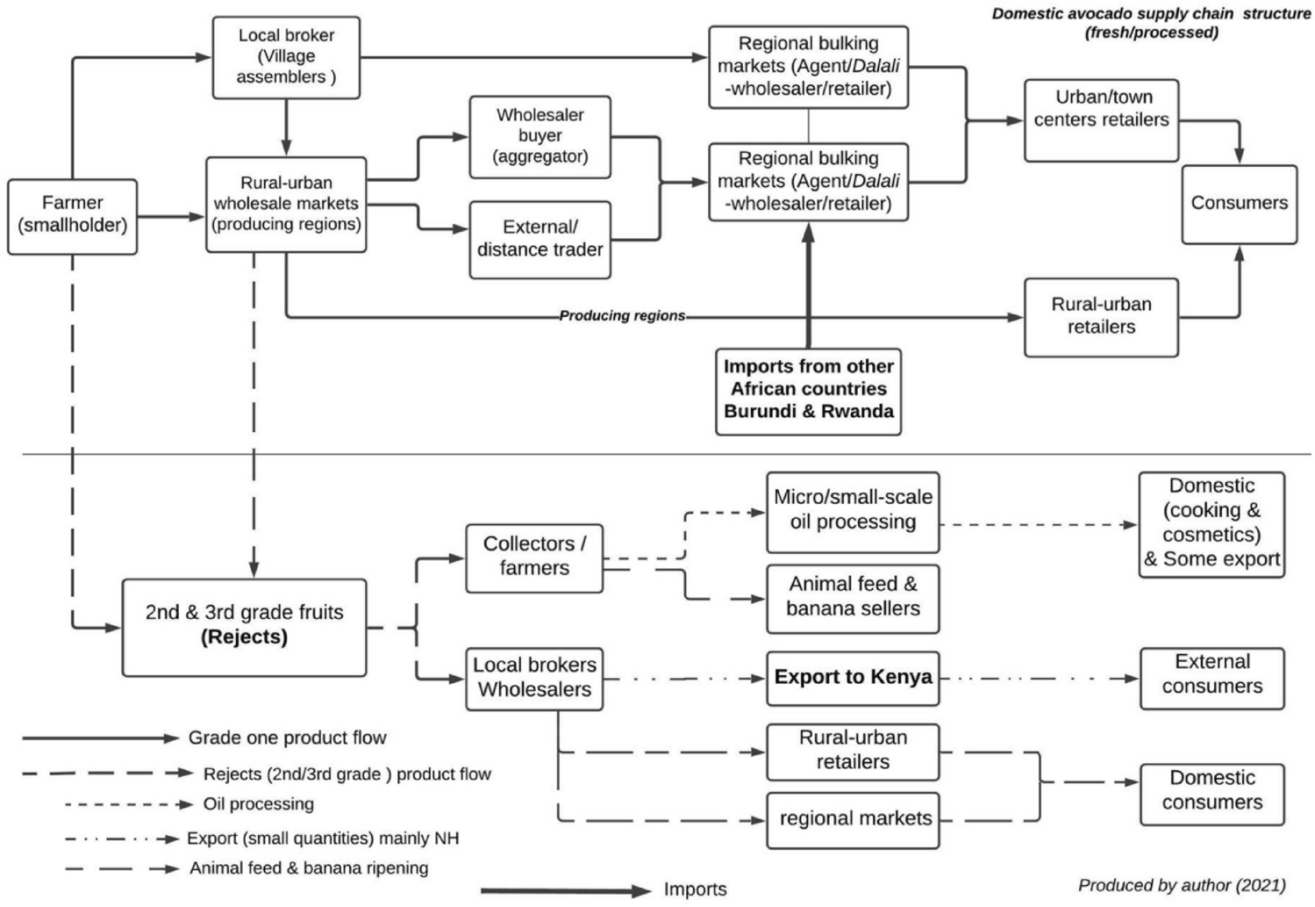
General overview of the domestic avocado supply chain. Source: interviews and observation

Figure 3 shows the structure of the domestic avocado supply chain. Farmers sell their produce directly to a local broker, a wholesale buyer, or rural and urban retailers. The crop is sourced either at the farm gate or in rural–urban wholesale markets (makeshift market points or gulio), which occur twice a week in the producing regions. While some farmers transport their produce to the markets, most sell to local brokers at the farm gate. There are three ways this happens. Usually, these are spot market transactions on cash terms (Eaton et al. 2008; Juma et al. 2019). Sometimes, farmers sell their fruits ‘on credit’ to local brokers with whom they have established long-term trading relations. In this case, the farmer is paid after the local broker has sold the product at the rural–urban wholesale market. Finally, on some occasions, forward sales are practised, whereby farmers receive part payment for their crop before harvesting or the fruit matures. Individuals and households are the primary market, with 65–70% of production sold by smallholders, 20% consumed on the farm (Mwakalinga 2014), and the rest used for animal feed and other purposes like ripening bananas (Cromwell 2022).

Local brokers are the primary assemblers who buy avocados from farmers and take them to the rural–urban wholesale markets. The local brokers, mostly women, dominate this stage of the supply chain and operate with limited capital compared to wholesale buyers, who are primarily men (Juma et al. 2019). Because local brokers are known in the areas where they operate, most transactions are based on personalised relations and trust with the farmer (Lyon and Porter 2010).

In major domestic avocado production regions like Kilimanjaro, Arusha, and Mbeya, most of the avocados are sold at rural–urban wholesale markets to wholesale buyers who aggregate the avocados from local brokers and farmers and transport them to regional markets in larger cities and urban towns. The agents (mostly in the regional markets) are responsible for selling the consignments delivered to the market by the wholesaler to retailers, consumers, restaurants, hotels, caterers, and street vendors. The structure of the supply chain is similar to other fresh fruit, vegetable, and spice supply chains in Tanzania (van der Maden et al. 2021; Akyoo and Lazaro 2007) in terms of its organisation, actors, and challenges. There is very little oil processing, due to the low oil content of the domestic avocado varieties. Even in the SH where there are oil processing factories, the factories only accept the export variety (Hass) which has high oil content.

#### Export supply chains

**Fig. 4:**
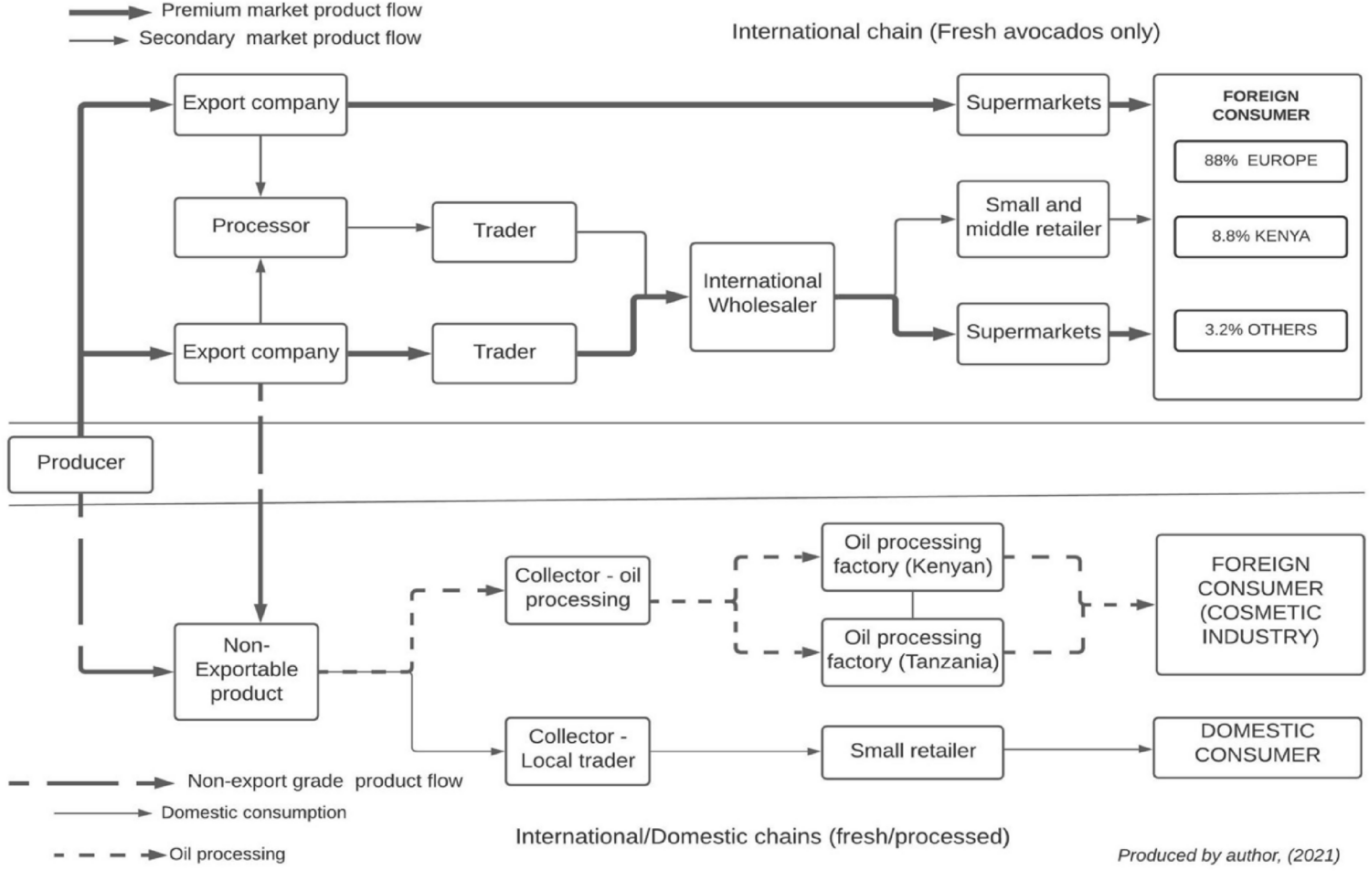
Structure of Tanzania export avocado supply chains. Source: Interviews and observations

Tanzania’s export avocado supply chain is structured into two chains (Fig. 4): (1) high-quality fresh avocados supplied through international supply chains; (2) low-quality avocados supplied through the domestic market (limited amount for human consumption) and for oil processing. Within the export supply chain, the dominant variety grown is the Hass, although other varieties (Carmen-Hass and Gem) are grown in the NH by two large commercial producers (124 hectares) under a special licence from Westfalia fruit International.

The high-quality fresh avocado export supply chain consists of ‘premium’ markets—where exporters supply directly to supermarkets across Europe; and ‘secondary or wholesale’ markets, in Europe, and other countries. The structure of the supply chain is similar to what has been identified by Barrett et al. (1999) in the Kenyan horticultural industry—export markets. They found that in the export market, two supply chains operate in Kenya, the wholesale supply chain, and the supermarkets supply chain.

In the case of Tanzania, the exporters operating the wholesale chain are largely a group of small and medium size packers/packhouses (Table 4), who have close relations with trading partners (importers). This ensures the flow of market information—quality, prices, and customer requirements (Bustos et al. 2018; Coronado 2010). These exporters supply mainly to the wholesale markets in the EU, UK, Asia, and to countries where certification and standard requirements are less strict.

**Table 4:** Typical characteristics of packaging houses

No. of packhouses	Capacity (tons/per)	Technology used/certifications	Main market destination	Company	Operational region
2	Large packers	Implemented Good Manufacturing Practices (GMP) Industry-specific standards/certification systems (Global G.A.P., BRC, GRASP, Tesco’s Nature, Albert Heijn protocol (AH), Sedex Members Ethical Trade Audit (SMETA) Computerised and automated cleaning, sorting, packaging, and refrigeration equipment Cold rooms for cooling/storing, and cold chain with atmospheric control containers No integrated farmgate cold chain Specialised personnel responsible for planning harvesting and production needs Trading department-vertically integrated with International Fruit trading and marketing company (Westfalia Fruits of South Africa)	Premium market in Europe (including the UK), Secondary markets in Europe, UAE, Middle East, China	Africado Ltd Rungwe Avocado Company (RAC)	Kilimanjaro Mbeya
3	Medium packers	Global G.A.P. certification Organic certification (some processors) Use mechanic equipment and develop activities manually Manual sorting and grading Cold rooms for cooling and storing No integrated farmgate cold chain transport Cold-chain transport Specialised personnel responsible for planning harvesting and production activities	Organic market (EU) Some premium markets (mainly EU) Secondary markets in Europe, UAE, Kenya, Zambia, India, Russia South Africa	Kuza Africa Ltd Four Seasons Orchard Ltd Tanzanice Agrofood Ltd (export their product as organic)	Mbeya Njombe Iringa
5	Small packers	Same as medium packers	The secondary market in Europe, Russia	Lima Kwanza Ltd Proganic Ltd Kibidula farm Ltd	Mbeya Njombe Iringa Iringa

Source: Interviews and observations

The premium or supermarket chains comprise mainly of large-scale commercial growers and exporters (e.g., Africado Ltd. and RAC), which have ‘fully integrated’ systems—production, processing, and selling is controlled by one company (Fuchs et al. 2009; Gereffi et al. 2005). These export producing companies have acquired the required Phytosanitary standards, private and supermarket-specific standards, and certifications (Table 4). Some export producing companies have more than six different certifications which allow them to supply directly to different supermarkets and retailers across Europe. Supplying directly to supermarkets offers premium prices, but occasionally these companies also supply the wholesale markets when market conditions are good, and during peak seasons or when there is a bumper harvest.

The large-scale farmers in the premium market chain make use of modern technologies (such as irrigation systems, fertilisation regimes, high-end chemical inputs, integrated pest management, soil, water, and leaf analysis) to achieve high yields of uniform-quality produce, as demanded by the supermarkets. Within the export chain, there exist “partially integrated” chains—these are large-scale independent producers (e.g., Kibidula Farm Ltd, Lima Kwanza Ltd) who have set up small-scale packhouses to export their produce but also to buy from smallholder growers.

**Fig. 5:**
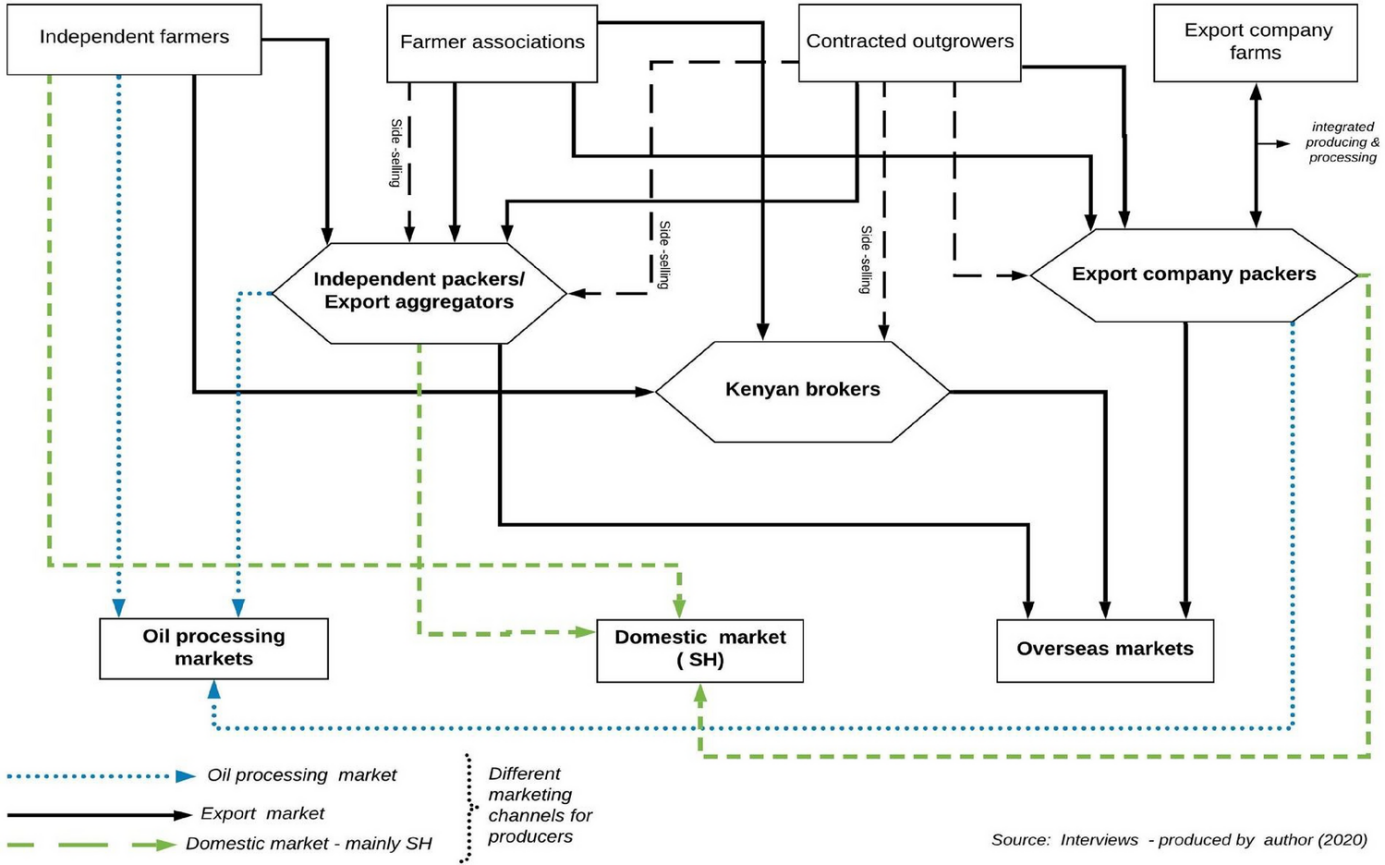
Marketing channels for export avocados. Source: Interviews and observations

The second sub-structure of the export avocado supply chain involves the supply of non-exportable fruits to the domestic market for oil processing and consumption. Produce that enters the domestic markets are rejects (from harvesting, sorting, and packaging) that do not meet export standards due to size, shape, colour, and appearance (blackened fruits due to over maturity or pest and diseases). In terms of oil processing, before 2016, rejected fruits from farms and packhouses in the NH and SH were dumped, leading to high volumes of food waste, with only small quantities donated to institutions such as schools and prisons. In 2016, the processor in the NH started exporting un-exportable fruits to Kenya for oil processing although the market is unsustainable due to lack of capacity. In the SH, in 2019/2020, three (3) oil processing factories started operating—buying non-exportable fruits from farmers and exporters. However, only a limited amount of non-exportable avocados is traded in the domestic market for consumption (in the SH) due to the lack of general acceptance (small size and rough appearance) of export variety (Hass) in the domestic market (Fig. 5). Consumers prefer larger-size avocados with smooth skin (domestic varieties), and this perception is culturally ingrained in the domestic avocado supply chain, because larger fruits provide value for money to the consumer: “with a large family size, if a consumer buys just one or two fruit(s), the whole family can share” [PTSH_DAO_0193].

### Practices, materiality, and institutions

Across domestic and export supply chains, we find that interactions between the practices, materiality and institutions that comprise these chains influence where loss and waste are created and who bears the burdens of this waste. In the following sections, we draw on analysis of agricultural practices, access to resources, handling and packing practices, quality assessment, norms, and contractual relationships to highlight a variety of ways in which unequal relationships between adjacent supply chain actors manifest in the distribution of waste-related burdens and risks.

#### Changing agricultural practices and norms

Export avocado production involves a significantly different set of agricultural practices compared to domestic avocado production. This is evident across irrigation regimes, soil management, and harvesting practices. Such practices have significant implications for loss and waste at the earliest stages of the supply chain. Loss of potential yield among local smallholder avocado producers is estimated at 50% due to flower and fruit drop (Mwakalinga 2014), which in turn is directly connected to soil nutrient and moisture content, which controls stress on the tree during fruit development (Bender 2012). The extent to which this loss is mitigated against through agricultural practices is inevitably reflective of the value of the avocado down the supply chain as well as the regulatory environment within which production takes place.

In the NH, smallholder farmers compared the value of the domestic avocado to that of the export avocado to explain the agricultural practices that are enacted in the two production systems.

The old varieties [domestic/local avocado], they are now marketable, but its market does not pay […]. We do not grow [plant] them, and you do not put manure, mulching or irrigate it; it just grows by itself. You see that it is scattered on the farm. When someone comes and says we need the avocados, they buy it [...]. However, the price they pay you is very low, so we do not take [see] it as a business. Because the tree is there, you just sell it [Female, smallholder, aged 67; PTK_ELF_0101].

According to export commercial farmers in the SH and NH, as part of site preparation, soil and water analysis are needed to determine macronutrient requirements and to decide on pre-planting soil treatment as well as to establish the site history, as explained by a farm manager:

Before planting, we take soil samples and send them to a lab for analysis... If the pH is normal but the Calcium is low, we add Gypsum. The soil analysis allows us to know pre-planting treatment needed, whether we add agricultural lime, Gypsum, rock phosphate, or something else. Once these additives are added in, then the seedlings are planted, and the irrigation system is put in [Male, farm manager; PTK_FM_0096].

Undertaking site, soil, and water analysis and pre-planting treatment comes at costs that are unaffordable to smallholder farmers and most independent medium and large-scale farmers. Thus, the majority of growers do not benefit from pre-planting soil treatments, which could improve production, increase yield, and reduce losses.

Traditionally, agricultural production among smallholders in Tanzania is rainfed. When additional irrigation is required, farmers irrigate using river/stream water, wells, and occasionally tap water. However, under Global G.A.P. requirements, farmers must irrigate with water that is laboratory tested. In the NH, most farmers reported that they are required to irrigate with tap water and must follow irrigation regimes specified in the growing manual. Undertaking irrigation practices among smallholders and medium-scale farmers involves significant farm labour (farmers irrigate by hand using buckets), known-how, and cost. Smallholders in both the NH and SH expressed frustrations and challenges they face enacting irrigation practices:

I have tap water, but I cannot afford it, if I have to use tap water to irrigate, the bills will be too high for me to pay [..]; the weather has not been very favourable to those who cannot afford water; if you can afford water, that is excellent [Male, smallholder; PTK_EF_0102].

When the tree has flowers, you must irrigate the tree twice a week so that the tree does not lose the flowers. Also, when the tree has many fruits, you must irrigate so that the fruit does not drop. Even if the fruits do not drop, they will be very small in size, shrink and turn black, and not grow well. For farmers who already have tap water at home, it is easy, but sometimes the day you want to irrigate, the tap water will not be available [Male, smallholder; PTK_EF_0074].

Access to water is a key issue for export avocado farmers compared to those growing the domestic varieties. Domestic varieties require less water compared to the export variety (Wolstenholme 2002), and because of the low economic value, farmers do not see the need to irrigate. Export farmers reported that water shortages and high temperatures can cause heavy flower and immature fruit drop (see Juma et al. 2019; Krymalowski et al. 2015). Losses are even more significant for farmers in lowland and midland altitudes because of higher temperatures and increased water demand. High fuel costs and the unreliability/unavailability of electricity in some remote areas inhibits the use of pumps for well irrigation (Ekka and Majwa 2020).

Besides irrigation practices, growing export avocado requires following a regime of fertilisation and pesticide management modelled on the tree phenology to ensure a healthy and a productive tree. However, the application of fertilisers and pesticides requires practical knowledge and technical know-how, and must follow strict rules and regulations to ensure safety and quality requirements are met, as indicated by an export manager and farm manager:

Yearly, before nutrients and fertilisers are applied, we do soil, leaf and water analysis using a recognised laboratory. The laboratory results are then sent to an agronomic consultant—Westfalia Technical Services in South Africa, which then provides recommendations and guidelines of fertilisers and micro-nutrients to be applied [Male Export manager, PTSH_TMGR_0207].

There are significant economic risks associated with conforming to the recommended agricultural practices of commercial exporters for out-growers. In the SH, some export companies provide input loan contracts to smallholders where they supply fertilisers and undertake fungicide/pesticide spraying activities on behalf of the growers, and the cost is deducted from the farmers’ harvest after sales. However, the “need to apply different nutrients to a single tree many times (7 or 8 times) increases labour cost” [PTSH_TMGR_0220]. Thereby disincentivising smallholders and some large-scale growers with limited knowledge and understanding of the quality requirements in the export market from undertaking fertiliser/nutrient and pesticide management activities that improve quality, thereby increasing losses and waste generation.

In the NH, chemical fertilisers and pesticides used among smallholders in out-grower scheme are prohibited. However, organic fertiliser alternatives, such as farmyard manure, which farmers are encouraged and allowed to use, are expensive for most farmers. The cost of manure can be more than TZS 1.4 million (612 USD) to fertilise an acre of orchard, and do not provide all the 12 nutrients required for a healthy productive tree (Gentile et al. 2016). Therefore, the production systems expose farmers to increased risk of crop pest and disease damage, resulting in losses and waste. Only producers that can take such economic risks are ultimately able to enter into such commercial out-grower arrangements.

#### Practices of handling and packing and the material nature of the avocado

Across domestic and export supply chains, practices of handling and packing avocados change through the supply chain and as the material nature of the avocado changes. Such practices are largely motivated by extracting as much value from the commodity as possible at any given stage in the chain, despite the trade-offs, which we consider as waste externalities, that are created further down the chain.

Within the domestic avocado supply chain, an accepted norm among traders, fruit pickers, packers, and loaders (irrespective of gender) is that the unripe avocado *“is as solid as a rock,”* which justifies packaging and handling practices such as pounding, standing, sitting, walking, and jumping on avocados during sorting and grading, packaging, loading, and off-loading at the farm and rural–urban wholesale market. Local brokers try to get as many avocados into a sack bag at the farm to increase their profit margins, through shaking, lifting, and pounding the sack bag on the ground several times during packaging. The practices are the same in the rural–urban wholesale markets when sorting and grading, as explained by different actors in the quotes below:

When packing, we ensure that a lot of avocados can go into the bag, we fill the bag well so that it is solid and compact. So, that when avocados reach the market in Dar es Salaam, the quantity will be the same” “We jump on the avocados while loading it into the truck, to makes it compact because we pack the bags in the middle of the truck” [...] [Loader and packer, Sanya Juu market, PTK_LB_0067]. “And jumping on the avocado does not cause any damage to the fruits [Female wholesale buyer, 14 years in the trade, WS_0068].

These practices are perpetuated and reproduced because of traders’ desire to extract more value through the process. However, they cause mechanical damage to the fruits that is only evident further down the supply chain as the fruit ripens (Kassim et al. 2013). The fragile nature of the avocado is concealed in its firmness at harvest and only becomes evident as its firmness decreases with ripeness—usually at regional and retail markets.

By contrast, avocados are handled with care at regional markets, traditional bamboo baskets (*Tenga*) and polythene bags used to pack fruits for customers are lined with dry banana leaves. At these later stages of the supply chain, the damage implications of mishandling are more immediately evident to adjacent supply chain actors (i.e., consumers) and therefore internalised by retailers at the point of sale. Thus, norms about the material state of the avocados’ firmness influence different handling practices by actors at various stages of the domestic supply chain.

Conversely, in the export supply chain, harvesting and handling practices require observing strict protocols-washing of hands with non-scented soap, wearing clean clothes, fingernails short, no eating, no drinking, smoking on the farm, and no wearing of earrings by farmers and fruits pickers. Such protocols ensure that fruits that come to the packhouse are free from foreign scent/taste but also to reduce bruises and scratches to fruits, which lead to losses during grading.

However, in enacting the harvesting activity, the materials that are mobilised and the practical know-how of farmers and fruit pickers interact together to generate losses. In most cases, farmers depend on household labour or hired labour to accomplish the task of harvesting and handling. Often, these labourers are drafted in on the day of harvesting and therefore lack practical understanding of using harvesting equipment, handling of fruits and the quality required, which leads to rough handling and inappropriate use of materials, causing damage to the fruits, as explained by a grower:

The challenge is how to harvest, not all workers know how to harvest these avocados. You try to explain and tell them what they must do but the head is not there [difficult to comprehend], they just can’t understand. That is a problem […], you have to train them, if you do not train them there will be blunders, you are going to regret it. You are going to cry, because you take the avocados to the packhouse, and all the fruits will be rejected. [Male, medium-scale grower; PTK_EFMS_0169].

Again, inadequate harvesting materials and lack of access to appropriate harvesting materials (like clippers, picking poles, and crates) mean farmers resort to using makeshift materials like buckets, baskets, and sack bags to complete the task of harvesting. Over-filling of crates (when available) causes mechanical damage and bruises, leading to rejections at the packhouse. However, on some occasions, even when farmers have access to harvesting equipment, they do not used them because: (1) they are in a hurry to complete harvesting tasks due to the limited time allowed for harvesting by export companies. Farmers complained that using picking poles slows the harvesting process; (2) sometimes, the picking poles are too short to reach the fruits, particularly where the trees have not been pruned, as is the case for most smallholder out-growers in the NH:

Another challenge during harvesting is that because of the shade and lack of pruning, some of the trees have grown tall; trying to pick the fruits that are on top of the trees is very difficult; that is why I have got this ladder to help with the picking, it is a very tedious job. We must look for helpers, who are very sharp in picking the fruits and can do it very quickly. Otherwise, the collection of the fruits will be delayed. [Male smallholder, lead farmer; PTK_ELF_0101].

The availability of appropriate technology and equipment for harvesting alone does not reduce losses. Rather, it is the interaction between technology access and other contextual and material factors, such as practical understanding and know-how, the planning of the harvesting schedule, and individual farmer circumstances that generate losses. Notwithstanding, the standardisation of products through quality standards especially those relating to aesthetic appearance—size, shape, and colour were found to result in large quantities of perfectly good fruits rejected at the packhouses. Sometimes, packers apply stringent quality criteria, to ensure perfect fruits for export and to reduce their own risk. Thus, the globalised export production, is embedded with regulations and standards that are in themselves waste-generating, and this is exacerbated sometimes by bad behaviour and practices of actors in the chain (Busch 2000, 2004; Freidberg 2004; Beausang et al. 2017; Johnson et al. 2019).

In the domestic production system, there are no extension services. Each ward is assigned one government extension officer responsible for all farmers, but some wards lack these officers. Additionally, these officers generally have limited knowledge about avocado production, making them unable to support farmers effectively (Juma et al. 2019). However, with the shift to export-oriented production, some local governments, such as Rungwe and Njombe Town Council, provide training to farmers. Most smallholder export producers receive their training through field officers employed by exporters as part of their contracts or through the farmer cooperatives/associations, although this training is often inadequate. Development agencies like the USAID, TechnoServe, and NGOs like TAHA have also played a role in the training of farmers and development of the export markets. Lack of adequate extension services in the avocado production system and horticulture sub-sector is key challenge that is recognised within policy discourse (ASDII and NHDS & AP 2021–2031). The lack of extension support services has been reported as a significant constraint to smallholder production in other export avocado-producing countries in East Africa (Lutta et al. 2024).

#### Different institutions and practices of quality checking, rejections, and pricing in the domestic supply chain

Quality checking and pricing institutions and practices across the domestic supply chain are reflective of the relative agency of, and social relations between adjacent actors in the supply chain. As indicated in Table 5, a variety of informal quality criteria and standards are applied by brokers, wholesalers, agents, and retailers, in domestic supply chain, typically focused on fruit maturity, size, eating quality, and appearance.

**Table 5:** Criteria used by traders to define and describe quality standards in the domestic avocado supply chain

Quality criteria	Local brokers’ quality criteria	Wholesale buyers’ quality criteria	Agents’ quality criteria	Retailers’ quality criteria
Maturity	Depending on the variety, the skin colour must change from shining green to dull/pale green or dark green, reddish, or purple. Shining green skin colour indicates immature fruit It should be easy to pop off stem/stalk from the fruit When you shake the fruit—you should hear the sound of the seed The seed coat is dry, dark, and somewhat shrivelled, not pale whitish and attached to the flesh The inside colour of the fruit flesh should be yellowish-gold or yellowish white Must ripen within 3–7 days after harvesting	Depending on the variety, the skin colour must change from shining green to dull/pale green or dark green, reddish, or purple. Shining green skin colour indicates immature fruit The inside colour of the fruit flesh (mesocarp or pulp) should be yellowish gold or yellowish white	The colour of the flesh (mesocarp) should be yellowish gold or yellowish white, not pale white Depending on the variety, the skin colour must be black or brown when the fruit is ripe Evenness in ripeness/firmness Must be ripe within 3–5 days	Depending on the variety, the skin colour must be black or brown when the fruit is ripe Evenness in ripeness/firmness
Size	Bigger sizes preferred Medium size Small sizes—low season	Bigger sizes first grade Medium sizes—second grade Smaller sizes only in low season	Bigger sizes preferred Medium sizes	Bigger sizes Medium sizes Smaller sizes only in low season
Eating quality (taste/palatability)	Medium to high oil content Flesh dryness/creaminess and smoothness High water content varieties are not preferred	Flesh dryness/creaminess is not considered significant if the fruits are larger sizes Eating quality is considered when buying medium or small size fruits	Consumers/buyers who know the different varieties and look at the eating quality smoothness/creaminess/good oil content Most buyers just buy based on the size	Medium to high oil content Creaminess—the avocado should be dry and not watery
Appearance	Good appearance, no blemishes Medium scratches/bruises Minimum visible cracks Avocado varieties where the skin remains green after-ripening are not preferred—varieties	Good appearance and attractiveness—can it attract a customer/buyer? Minimum scratches/bruises No visible cracks Avocado varieties where the skin remains green after-ripening are not preferred	Good appearance—little or no wrinkles, or shrivelled Not overripened Fruits must not shrivel and ripen abnormally (immature fruits do) The outer skin of ripened avocado must be brown or black or purple	Good appearance–little or no wrinkles, or shrivelled outside Not over-ripened Fruits must not shrivel and ripen abnormally (immature fruits do) The outer skin of ripened avocado must be brown, black, or purple

Source: Interviews and observations

The most common external indicators used to assess maturity level are “skin colour change, easiness to pop-off stem/stalk, and hearing the sound of the seed when you shake the fruit” [Female, local broker, PTK_LB_0024]. Eating quality is generally determined by flavour, texture (creaminess and smoothness), and oil content, which is influenced by fruit maturity, and the stage of ripeness (Magwaza and Tesfay 2015; Hofman et al. 2013). Traders check oil content through destructive method, by scrubbing a piece of fresh avocado on the back of their hand. Through this embodied practice, traders differentiate between varieties with a high-water content (so-called “watery avocados”), and varieties with high or medium oil content, which has implications for losses and waste. During peak season, there is high wastage for ‘watery avocados’ because of the perceived poor eating quality by consumers and therefore farmers struggle to sell these avocados.

Besides maturity and eating quality, traders use ‘fruit size’ as the final criterion for buying decision. Notably, among wholesale buyers, size tends to influence purchase decisions irrespective of the eating quality once an avocado passes the maturity criteria. Bigger fruit sizes attract better prices compared to medium or small sizes. The low value of small and medium-size avocados, especially in the peak season, makes it a high risk for local brokers to buy. An excerpt from the field journal during a harvesting participant observation event in the Kilimanjaro illustrates this:

On the first harvesting event, as we walked, one of the brokers pointed to an avocado tree and commented: “We have seen this avocado and its’ not good for us […], because of the size of the fruits we are not going to harvest it. Buyers from Dar es Salaam won’t buy; it’s not marketable” although the fruits were matured. On the second occasion, the brokers have approached the farmer and decided to buy the avocado; after picking a few fruits for quality checks, they decided not to harvest because of the fruit size: “If we buy it, it will be a loss to us, ‘if you do not have an order from a buyer, its’ impossible to sell small size fruits” [Field Journal, 18 June 2018].

As a risk avoidance strategy, traders apply stringent size criteria during the peak season, leading to higher losses and waste on farms and in the markets. However, determining what counts as a small, medium, or big ‘size’ is not a ‘static feature’ as there is no standardised measurement. Determination of the ‘size’ is a subjective judgement of the buyer. It is more fluid and varies among buyers and across different production sites/markets as well as the varieties of avocado themselves (different varieties have different fruit sizes and shapes).

Farmer’s price or measure their avocados through three modes: using a bucket *(debe),* sack bag, or pricing ‘per tree’ (the broker estimates the volume of fruits and negotiates the price). The method used for pricing determines who bears the losses associated with variability in quality. If the farmer agrees to price per sack bag or bucket, only the avocados that individually meet quality criteria are included in pricing. However, if it is priced per tree, the local broker takes everything. Brokers prefer pricing per tree for two reasons: 1) To avoid an argument with the farmer over price; 2) to extract more value since most farmers cannot accurately estimate how many fruits are on the tree as explained by a leading local broker in Kilimanjaro:

[…] It depends on what each broker is comfortable with. So, it needs experience; brokers who understand and are used to the situation and have been in business for many years will know exactly how to estimate; maybe from this tree, I will get this much. So, if I buy a tree, I will benefit; but I prefer to purchase either using sack bag or bucket. I know my profit and loss [Female, leading local broker, Rombo; PTK_LB_0042].

Inevitably, some farmers prefer ‘pricing per tree’ to create value—if they cannot derive other values (animal feed) from the rejected fruits (losses), and to prevent disagreement [‘ubishi’] over price. Disagreement over price was reported by farmers in the NH as the biggest challenge:

After you have agreed on the price with the buyer, they sometimes change their mind about the agreed price after they have harvested the avocados, which usually leads to argument. When this happens, the buyer will say, I do not want to buy the avocados anymore. And you have harvested the avocados; if you cannot sell, what are you going to do with the avocados? The avocados will decay, so you sell at any price. It is like they are exploiting you, to sell the fruits at any price [Male, smallholder, PTK_LF_003].

As avocados ripen once picked, there is a critical time window for selling before the avocados begin to over-ripen and therefore lose value. The result is that, once picked, farmers have limited agency when it comes to price negotiation and selling. The same is true for other adjacent actors in the supply chain, as expressed in the quotes below:

The wholesale buyers do not care if they buy your fruits or not. The relationship between you and the wholesaler does not matter; they always look at the avocados [quality standard]. Even if they decide to buy, you must accept any offered price. You have brought the avocados to the market; what are you going to do with the avocados? [Female, local broker; Sanya Juu market PTK_LB_0064].

I buy the avocados on credit from the wholesaler in Kilimanjaro and sell them myself [….]. In a month, I take delivery of about 100 bags. This is how the credit system work; because I do not pay the wholesaler, I estimate that if I get a profit of say 15,000 TZS [6.47 USD] per bag after all cost deductions, that is enough for me. After selling, I will negotiate with the wholesaler, I have not made any losses, and you have so much profit, so we must share the profit. In this way, I reduce the gain for the wholesaler […]. And because I take the avocados on credit, if there are any losses, it is for the wholesaler [Agent, Male, Temeke Sterio market, Dar es Salaam; PTD_TA_0092].

The inequalities within the social relations described above and their associated implications for waste generation is driven by profit and the need to extract value for adjacent actors in the value chain. While our study did not focus on a detailed analysis of profit margins and its variability, a compilation of farmgate and rural wholesale prices in two districts in Kilimanjaro region and in Dar es Salaam, indicates that the farmers are poorly paid. Price at the farmgate is between 10,000 and 22,000 TZS for a 150 kg bag, and at the wholesale market (production area), a 100 kg bag is sold for 30,000–60,000 TZS while the same 100 kg is priced at 50,000–75,000 in Dar es Salaam depending on the season. Profit margins vary depending on the season. In Tanzania, marketing margins for brokers along national agricultural produce supply chains range from 4 to 20% (Eskola 2005a, b). Similar margins have been reported among avocado brokers in Ethiopia. Shumeta (2010) noted that wholesalers in the Ethiopian trade gained 35.41% of the gross profit from the transaction compared to about 25% margin earned by brokers.

#### Contractual growing, security and lock-ins in export supply chains

The degree of formalisation in the relationship between supply chain actors can have important and sometimes conflicting implications for the distribution of risk and the burden of waste. It is common for small-scale producers to sell through informal and short-term trading relationships or spot buying arrangements that carry uncertainty in timing and pricing. Having an out-grower contractual relationship can provide a degree of price security and less exposure to brokers for producers, but they are also associated with risks of being ‘locked-in’ to the contractual relationship with severe sanctions if producers fail to follow the rules and guidelines set out in the production and marketing manual (Clapp 1994; Friedberg 2004).

The Global G.A.P. certification scheme requires smallholders to be organised into farmer groups, either as self-organised or through export company’s out-grower scheme. In this study, the export companies organise and manage farmer groups for certification, which means the exporters manage the “Quality Management System”—referring to the system put in place by the exporters to ensure certification scheme requirements for both internal and external audit are met (Holzapfel and Wollni 2014). The implication is that the export companies own the certification, and control and determine the production requirements for farmers. We found the most common certification scheme for farmers to access the international markets was global GAP. Although most smallholder production does not involve chemical inputs, the majority of farmers have not been certified as organic producers and therefore do not benefit from the premium price in the international market except for few farmers in the SH who have been have been certified as organic producers by a medium-scale exporter Tanzanice Agrofood Ltd (see Table 4).

Although we did not investigate contract schemes for organic certified farmers, price comparison across exporters in SH (Table 6) shows that farmers who sell their produce under organic scheme are not necessarily better off compared to farmers selling their products under different contract scheme/farmer associations. The promotion of organic certification or fair trade is very limited. While fair trade certification is somewhat common among tea and coffee farmers, the adoption of fair trade in avocado production does not exist.

**Table 6:** Export avocado prices in the NH and SH

Northern Highlands price Per Kg (Africado ltd) Prices are in Tanzania Shillings				Southern Highlands price per Kg (Exporters & Cooperatives) Prices are in Tanzania Shillings							
Year	Rombo District (smallholders)	Siha District (smallholders)	Large commercial farmers	UWAMARU AMCOS Rungwe	Rungwe Avocado Company (RAC)	KUZA Africa ltd	Lima Kwanza Ltd	Four Seasons Ltd	Njombe Avocado Farmers Network	Tanzanice Agrofood Ltd	Rungwe District floor price
2009											
2010	400	550	935								
2011	600	700	1190		250						
2012	680	824	1401		250	Buyers/processors not in operation					
2013	800	996	1693		270						
2014	1000	1029	1749		300						
2015	1133	1258	2139		607						
2016	800	1025	1743	600	750						
2017	1135	1200	1500–2000	1000	700	1000	700			1200	
2018	685	750	1200–3000	1200	1200	1200	1200	1200	1200	1500	1000
2019	1200	1320	?	1500	1450	1400	?	1500	1500	1500	1300
2020	750	900	?	1500	1250	1200	?	1600	1600	1500	?

Source: Interviews and payment records from farmer

We noticed some price disparities among growers in the NH, where large-scale farmers were paid higher price per kg compared to smallholders for the same quality of avocados. Generally, smallholders in the SH are paid higher prices compared to their counterparts in the NH (Table 6), this is due to competition among exporters. In the NH, there was only one exporter at the time of this study.

For farmers engaged in Global GAP schemes, we found that in some out-grower schemes, individual farmers are not engaged in a contract negotiation process, contract terms are set by exporters, and representatives of the farmer group sign. Most of the farmers interviewed in the NH did not have copies of a contract, except lead farmers/group representatives:

The farmers do not have a copy of the contract; they just sign the contract every year... The company say that the contract is not complete, every year they try to add something to the contract, so when you ask the company, why can’t we give copies to farmers, the answer is that if we give farmers a copy, how can we come back and take that copy and change it? [PTK-FO-0166].

The lack of access to a contract by farmers gives rise to a lack of transparency and inequalities in grower-purchaser relationships. In cases where the exporter enjoys monopsony power and controls the out-grower association, there are serious implications when farmers breach the contract, as expressed by most farmers:

As a group, we must not sell [our fruits] to anyone else apart from the company. Other buyers come from Kenya, but we are told not to sell to another buyer. If the company notice that you have sold your avocados to someone else…, they will not buy from you again, and you will be removed from the association [...]. One thing that most farmers fear is [that] if you breach the contract with the company, you cannot get a buyer from Kenya… just to come and buy your fruits as a [individual] farmer [Male, smallholder, lead farmer; PTK_EF_0077].

Therefore, a breakdown of contract, over which there are unequal power dynamics in the setting and security of, can be a significant source of waste generation. We found that as part of the Global GAP certification scheme, out-growers, especially in the NH have their harvested crops aggregated together during grading at the packhouse, and any rejections are shared proportionally among the group. This presents a significant source of inequality and waste generation opportunities as expressed in the quote below:

Last year [2017] I sold 290 kg, and I was paid 250 kg, so about 40 kgs was a reject. Sometimes you can be careful during the harvesting, but other farmers may not be careful and bring many rejects. Maybe you have a tiny [amount] of reject, but the company does not care about that; usually, all the rejects are added together and shared among the farmers according to the number of kilos the farmer sells to the company. If you have sold a lot of kilos, it means you will have a lot of rejects [Male, smallholder, PTK_EF_0077].

The institutional arrangement of ‘sharing rejects’ among smallholders causes losses and waste through opportunistic behaviour and bad practices (such as harvesting immature fruits and non-export varieties to increase their farmgate volumes) as farmers seek to reduce their risk. As in many of the materiality, practice, and institutional interactions described above, the burden of this waste falls predominantly on the producer, and there is significant risk involved in entering into agreements and supply chains that ought to provide more formalisation and security in cross supply chain interactions. Notwithstanding, participating in the export supply chain has provided opportunities to most of the smallholders (80–90%) who own less than ten productive trees to improve their livelihoods and view export avocado production as a viable business compared to domestic avocado and other agricultural crops albeit the numerous challenges and inequalities they face (Table 7).

**Table 7:** Prices of Avocado at the farm gate and wholesale markets (2019/2020)

Year (2019)	2019/2020 Avocado Prices in Kilimanjaro and Dar es Salaam (per 150 kg and 100 kg sack bag)				
	Farmgate price (Siha District) Per sack bag (150 kg)	Farmgate (Rombo Dist. Per sack bag) (150 kg)	Rural–urban Wholesale Market (Sanya Juu—Siha Dist.) Per sack bag (100 kg)	Rural–urban Wholesale Market (Mwika/Mamsera—Rombo Dist.) Per sack bag (100 kg)	Wholesale market in Dar es Salaam Per sack bag (100 kg)
Jan	15,000	15,000	50,000	50,000	70,000
Feb	12,000	13,000	45,000	45,000	70,000
Mar	10,000	10,000	35,000	40,000	60,000
Apr	10,000	10,000	25,000	30,000	50,000
May	10,000	10,000	30,000	30,000	50,000
Jun	10,000	10,000	35,000	30,000	60,000
Jul	10,000	12,000	40,000	35,000	70,000
Aug	12,000	12,000	45,000	40,000	70,000
Sep	12,000	13,000	45,000	45,000	70,000
Oct	15,000	15,000	50,000	50,000	70,000
Nov	15,000	15,000	55,000	60,000	70,000
Dec	20,000	22,000	60,000	60,000	75,000

## Discussion

In the themes that emerged from following and analysing avocado supply chains in Tanzania, it is evident that a focus on practices, materiality, and institutions helps to draw attention to the relative agency and power dynamics between adjacent actors in these supply chains. This manifests to some extent in how risks and the burden of waste are distributed.

A common theme is that the burden of waste falls heaviest on producers. They are producing a highly perishable commodity for markets in which there are both informal expectations and norms as well as formalised requirements for food quality. In the domestic avocado supply chain, producers operate within supply chains in which there is high dependency on brokers, agents, and wholesale marketers who apply a risk-averse approach to enforcing standards as well as their own norms (sometimes in the form of inconsistently applied quality checking and price setting) on their suppliers. A focus on risks reinforces how power and inequality work in food systems (Beck 1992). To reduce risk, the ‘perishability’ of the avocado is used as vehicle to exercise power by the actors along the value chain, and the exercise of power shifts as the avocado is exchanged along the chain. At the farmgate, brokers exploit farmers by using the fruit’s perishability to reject produce and force sales at low prices. Once the fruits have been harvested, farmers have to sell at all costs for any price. If disagreements over price lead to additional handling, this causes further damage to the fruits, leading waste generation downstream. Farmers’ remote locations and high transaction costs make them vulnerable, compelling them to sell to brokers who set lower prices. Similar exploitation is reported in other fruits and vegetable supply chain in Tanzania (Dube et al. 2018; Mayala & Bamanyisa 2018), and in domestic avocado supply chains in Kenya (Omolo et al. 2011), and Ethiopia (Megerssa 2013).

In rural–urban wholesale markets, buyers exploit the perishability of avocados to exert power over local brokers, frequently changing prices at short notice. This power dynamic has been noted in other fruit and vegetable markets in Tanzania (van der Maden et al. 2021; de Putter et al. 2007) and in domestic avocado supply chains in Kenya and Ethiopia (Omolo et al. 2011; Shumeta 2010). Brokers, with limited bargaining power, must sell quickly to avoid losses (since the fruits ripen within 3–5 days after harvest), often at a loss. If unable to sell to wholesalers, brokers face further losses selling in local retail markets with lower demand. This power imbalance leads to significant food losses and waste, loss of income, but also loss of economic days as the brokers must spend several days to sell in small quantities at local retail markets.

In regional markets, agents prefer the credit system to reduce risks and pass them to wholesalers. Rapid changes, such as over-ripened avocados or bad weather, allow agents to exploit wholesalers. Established norms about causes of losses determine who bears the associated costs. These established norms weaken the wholesalers’ position, makes them vulnerable, and allow agents to perpetuate inequalities against them. If an agent bears the cost of losses, the wholesaler must continue supplying to keep the agent in business, thus ‘enslaving’ the wholesaler, especially, if the loss involves a considerable sum of trading capital. Lyon and Porter (2009, p. 910) noted that credit suppliers to vegetable traders in Nigeria’s Jos Plateau lack power, and pressured credit receivers can use local moral judgments to avoid repayment responsibilities.

However, there are also some cases of actors pushing the burden of waste further down the supply chain. For example, brokers’ and wholesalers’ handling practices, packing as much fruit as possible into sack bags, reduce transportation and storage-related costs at that point in the chain, but results in bruises and mechanical damage to fruits, which causes losses and waste further down the supply chain (markets in Dar es Salaam and other cities). Practices and relationships across the supply chain are often dictated by attempts to maximise value and push costs onto others, and the ability of different actors to mitigate waste in this context depends in large part on the material nature of the avocado.

In the export supply chain, the enforcement of supply chain regulation can be a force for empowering producers through protecting fair trade and worker’s rights, for example, and as such, entering into more formalised commercial and export markets might be expected to be associated with improved economic security and agricultural livelihoods. However, we find that in the case of food quality regulation, this does not only places additional burdens and costs of compliance onto producers, who are primarily responsible for meeting Global GAP or retailer regulations, but it is also disempowering in the sense that they become a means for justifying price reductions, sanctions, and fruit rejections as contracts are used as risk avoidance strategy by which exporters transfer production risks to farmers (Little and Watts 1994; Mazwi 2020). For these reasons, entering into commercial export supply chains, often through participation in farmer associations, carries significant risks for producers who may find themselves with several lines of dependency—not only on contractors, but also on agro-input suppliers and on the other producers that make up their association. Resource-constrained, low-income farmers may be those least able to take risks and to absorb the economic costs of compliance and of loss and waste. A detailed discussion on the institutional arrangements, types of social relations, and how power inequalities lead to waste production in the export avocado supply chain is covered elsewhere.

It is critical to reduce food loss and waste within both the domestic and export avocado supply chains to improve food and nutrition security and the livelihoods of actors along the value chains. With 65% of the Tanzanian population depending on agriculture for food and employment and supporting the livelihoods of over 80% of rural agrarian families, reducing food waste will enable Tanzania to achieve its vision of ‘ensuring that all Tanzanians will have access to healthy diets and safe foods, focusing on life-cycle, and address all forms of malnutrition by 2030’ as set out in the National Road Map for Sustainable Food System Transformation (URT 2021a). Food loss and waste represent a loss of resources used in the production, processing, and distribution but also a loss of income to farmers, processors/exporters, and traders (FAO 2019); which not only impacts livelihoods but also risks reducing poverty, especially among the rural poor who are engaged in production activities.

By integrating institutions, materiality, and practices into one framework, this study approached losses and waste from a social relations perspective (Gille 2010, 2013), in contrast to more conventional studies of waste as a technical challenge. Thus, moves food loss and waste debates beyond a collection of isolated views on a particular unit of analysis (e.g., modelling, auditing, and quantification of loss and waste and drivers) towards a deeper understanding of the complex causes and implications of agricultural losses and waste. Several studies focusing on consumer food waste in the global North (e.g., Evans 2011, 2012, 2018; Watson and Meah 2013) point out that household food-wasting practices arise from complex contexts: socio-cultural, economic, food safety concerns and anxiety, and social relations that are deeply entangled with everyday routines. Further, this study extends the sociological gaze on losses and waste with a global South perspective, where such approaches are lacking in academic debates. It therefore makes significant contributions to wider critical food waste literature, more specifically to the food waste regimes concept (Gille 2013), and pushes against the idea of identifying food loss and waste in the global South as only associated with inadequate infrastructure, identifying it instead with a myriad of activities from which losses and waste emerge.

Moreover, the study demonstrates the ways in which the distribution of loss and waste can itself be illustrative of unequal social relationships (Moreno et al. 2020). The implication is that tackling food loss and waste and tackling social inequality are one and the same challenge. In addressing this interlinked challenge, we would call for greater formal and informal efforts:

1. Local government and central government institutions should work closely with exporters and processor, to promote flexibility and supply chain engagement for producers. In this regard, bottom-up farmer associations and cooperatives like UWAMARU, Njombe Avocado Farmers Association, and Mbeya Avocado Farmers Association (MBEAFA), and contracted out-grower schemes like Muviwapasi Association, should be strengthened to provide the needed training and extension services on production, harvesting, safety and quality, to their members but also to offer flexibility to farmers of which exporter to sell to. For instance, MBEAFA is a collection of small farmer groups within the Mbeya region and works with several exporters. This offers farmers the flexibility to engage with any of the exporters the association works with.
2. Local governments in the production areas should implement and strengthen safeguards against exploitation, especially price exploitation, and in the way input loans supplied to farmers are paid back. For example, the Rungwe district local government at the start of the harvesting work with exporters to set the minimum price (Table 6). This ensures that no exporter can sell below the floor price.
3. Local governments and exporters, especially those that have contracts with farmers, should make up-to-date market information easily accessible to farmers, including the costs associated with processing and exporting. In the NH, farmers in out-grower schemes sell their produce without knowing the price at which they are selling until the processor has sold the product at the international market. Ensuring price transparency will reduce disparities and inequalities in the system
4. The local government should promote consistency in food quality regulations and standards across processors and exporters to ensure that the right standards are applied across the board to all farmers. This will help reduce the level of rejections.
5. Local governments, development agencies, NGOs, and organisations like TAHA should offer advisory services for contract negotiations and management and ensure transparency for all in contractual agreements.
6. In the domestic supply chain, market associations, market managements, along with local extension officers, should train farmers, local brokers, and wholesale buyers on poor fruit handling and value extraction practices that lead to inequalities and waste generation.

There are likely to be commonalities across different contexts and commodities, and these general principles for addressing food waste and social inequity should be broadly applicable. However, the relevance of materiality, institutions, and practices in our research, also helps to bring to light the contextual nature of food loss and waste challenges. In this case we have considered a highly perishable commodity that ripens after harvest, a commodity for which the characteristics valued in local markets are different to those valued in export markets, and a commodity with supply chains that have significant informal and formal characteristics for distinct markets. As such, the critical windows of opportunity for intervention (and the points in the supply chain at which exploitation takes place) may be quite different for other commodities in other contexts. We therefore argue for the need to build on emergent efforts in exploring food waste regimes and social relations across diverse food supply chains and to share and compare findings across this work.
